# Prediction and validation of potential transmission risk of *Dirofilaria* spp. infection in Serbia and its projection to 2080

**DOI:** 10.3389/fvets.2024.1352236

**Published:** 2024-04-03

**Authors:** Iván Rodríguez-Escolar, Ricardo Enrique Hernández-Lambraño, José Ángel Sánchez-Agudo, Manuel Collado-Cuadrado, Sara Savić, Marina Žekić Stosic, Doroteja Marcic, Rodrigo Morchón

**Affiliations:** ^1^Zoonotic Diseases and One Health Group, Biomedical Research Institute of Salamanca (IBSAL), Faculty of Pharmacy, University of Salamanca, Salamanca, Spain; ^2^Biodiversity, Human Diversity and Conservation Biology Group, University of Salamanca, Salamanca, Spain; ^3^Center for Environmental Studies and Rural Dynamization (CEADIR), University of Salamanca, Salamanca, Spain; ^4^Scientific Veterinary Institute "Novi Sad", University of Novi Sad, Novi Sad, Serbia

**Keywords:** *Dirofilaria* spp., infection risk, ecological niche modeling, *Culex pipiens*, projection, Serbia, Europe

## Abstract

Animal and human dirofilariosis is a vector-borne zoonotic disease, being one of the most important diseases in Europe. In Serbia, there are extensive studies reporting the presence of *Dirofilaria immitis* and *D. repens*, mainly in the north of the country, where the human population is concentrated and where there is a presence of culicid mosquitoes that transmit the disease. Ecological niche modeling (ENM) has proven to be a very good tool to predict the appearance of parasitosis in very diverse areas, with distant orography and climatologies at a local, continental, and global level. Taking these factors into account, the objective of this study was to develop an environmental model for Serbia that reflects the suitability of the ecological niche for the risk of infection with *Dirofilaria* spp. with which the predictive power of existing studies is improved. A wide set of variables related to the transmission of the parasite were used. The potential number of generations of *D. immitis* and the ecological niche modeling method (ENM) were used to estimate the potential distribution of suitable habitats for *Culex pipiens*. The highest probability of infection risk was located in the north of the country, and the lowest in the southern regions, where there is more orographic relief and less human activity. The model was corroborated with the location of *D. immitis*-infected dogs, with 89.28% of the country having a high probability of infection. In addition, it was observed that the percentage of territory with optimal habitat for *Culex* spp. will increase significantly between now and 2080. This new model can be used as a tool in the control and prevention of heartworm disease in Serbia, due to its high predictive power, and will serve to alert veterinary and health personnel of the presence of the disease in the animal and human population, respectively.

## 1 Introduction

Vector-borne diseases have a significant negative impact on both animals and humans worldwide (1). One of the most important factors to consider is anthropogenic global warming, which has led to changes in the composition of terrestrial and coastal ecosystems, one of the main causes being the increase in temperature and the consequent spread of new vector species to previously vector-free areas (2–4). In the case of Europe, moreover, the increase in the intensity of human activity, as well as new agricultural methods and the expansion of irrigated cultivation, has led to a substantial increase in countries close to traditional endemic countries such as Portugal, Spain, France, Italy, Greece, and Turkey (5–7).

Dirofilariosis is a worldwide vector-borne zoonotic disease and one of the most important animal diseases in Europe. *Dirofilaria immitis* and *D. repens* are the most important causative agents of the disease in its definitive hosts, which are domestic and wild canids and felids. The domestic dog is the main reservoir or the one for which most data are known, and its vectors belong to the genera *Culex* spp. and *Aedes* spp. and are widely represented throughout the European continent (7–12). Humans act as accidental hosts, coming into contact with the parasite more frequently in places where microfilaremic reservoirs exist, which can lead to human dirofilariosis (10).

In Europe, changes in its distribution pattern have been documented, with most countries being endemic with a broad change in the last 20 years (7, 10, 13). The distribution of the disease is favored by the presence of vectors, as well as with the presence of fresh water, high humidity, and average temperatures. When the environmental temperature increases, the period in which the larvae mutate inside the vector is shortened (14, 15).

In Serbia there are several studies that report the presence of cardiopulmonary dirofilariosis in dogs, being 3.17–16.1% in the north, in the capital (Belgrade) 22.01%, even with coinfections with *D. repens* in 3.97% of the dogs, and in Kosovo 9% (16–21). In recent years, prevalences in dogs have increased in the north of the country, with ranges between 12.7 and 33.3%, together with the presence of some microfilaremic dogs and in the south (Kosovo) with prevalences due to *D. immitis* of 14.8% (20, 22–25). In addition, studies of the presence of *D. immitis* in wild animals such as gray wolf and red fox, golden jackals, and wolves have been reported with prevalences between 1.55–7.32 and 7.79% in wild cats (26–28) and for the first time, the presence of *Dirofilaria* spp. in three species of culicid mosquitoes: *Cq. richiardii*, *Cx. pipens*, and *Och. caspius* (29).

Ecological niche modeling (ENM) has proven to be a very good tool in predicting the occurrence of parasitosis in very diverse area, with distant orographies and climatologies at local, continental and global levels (30–38). These models are based on the processing of robust environmental and bioclimatic variables, as well as others directly related to vector, and thus assess the probability of transmission of vector diseases (5, 39–43). One of the most important models for this situation and one of the most widely used is the maximum entropy algorithm (Max-Ent), which uses presence data and produces robust and very accurate statistical models (42, 44–46).

In Serbia there are no specific investigations that have allowed predicting the risk of *Dirofilaria* spp. infection, but there are studies (5, 47) for the European continent that incorporate cartographic information in their spatial analysis with GIS temperature records. However, there are no studies for Serbia that take into account orography, climate, environment, human activities or population centers, among others. Considering that with ENMs it is possible to relate the presence of a zoonosis to biotic variables, extrapolate it to other areas without vector presence data and know its dynamics over time at high resolution, as well as take preventive control measures to avoid the expansion or eradication of a zoonosis, the arm of this study was to develop an environmental model for Serbia that reflects the suitability of the ecological niche for the risk of infection by *Dirofilaria* spp., taking into account, in addition to the average annual temperature, other bioclimatic and environmental variables, and the number of generations of *Dirofilaria* spp. that can be developed in the vector, as a novel contribution that improves the predictive models carried out at the European level, improving their resolution and significance.

## 2 Methods

### 2.1 Description of the study area

**Figure 1:**
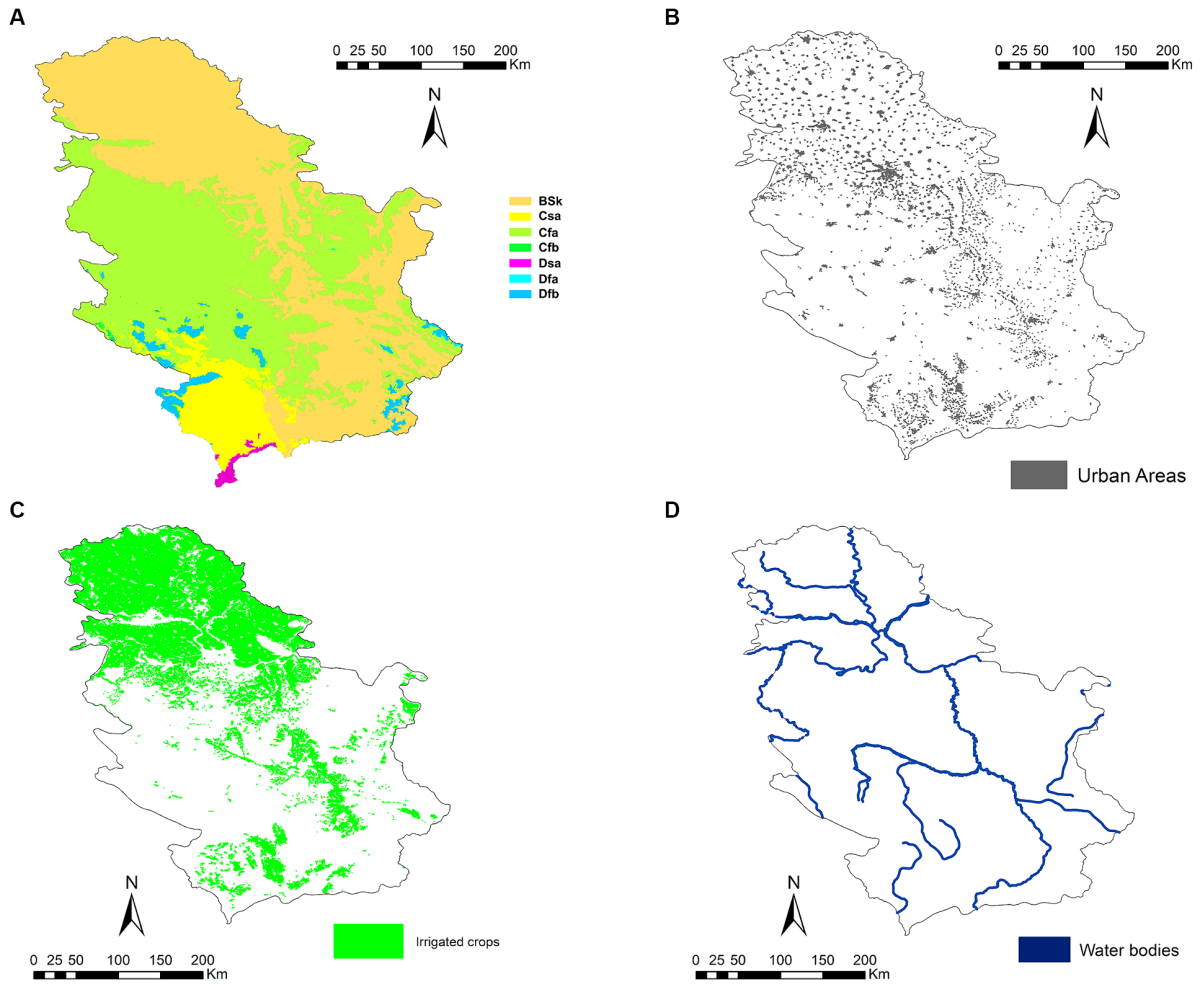
**(A)** Climates according to the Köppen Climate Classification System (BSh: hot semi-arid climate; BSk: cold semi-arid climate; Csa: hot-summer Mediterranean climate; Csb: warm-summer Mediterranean climate; Cfa: humid subtropical climate; Cfb: temperate oceanic climate; Dsb: humid continental climate; Dsc: subarctic climate; Dfa: hot-summer humid continental climate; Dfb: humid continental climate; Dfc: subarctic climate; and ET: Tundra), **(B)** human populations, **(C)** irrigated crops, and **(D)** water bodies in Serbia.

Serbia (44°0′59.5″ N 21°0.352′ E) is a country in southeastern Europe located on the landlocked Balkan Peninsula, bordered by Hungary to the north, Romania and Bulgaria to the east, North Macedonia and Albania to the south, and Bosnia and Herzegovina, Croatia, and Montenegro to the west. The province of Vojvodina, in the northern third of the country, is part of the Central European Pannonian Plain. The rest of the country is mountainous, with the Dinaric Alps in the center, west, and southeast. The easternmost part of the country is the Wallachian Plain, while the western border is determined by the Carpathian Mountains. The Southern Carpathians meet the Balkan Mountains in the southeast of the country, following the course of the Great Morava River. Most of Serbia’s territory (92%) belongs to the Danube River basin, which dominates the north of the country. Besides the Danube, the main rivers are its tributaries the Sava (coming from the west), the Tisza (coming from the north), the Drina (coming from the south) and the Morava, the latter flowing almost entirely through Serbia in the mountainous southern regions. Due to the geography of the terrain, natural lakes are few and far between, but there are numerous bodies of water of artificial origin. The country’s climate is continental, alternating between a Mediterranean climate influenced by the Adriatic Sea in the south with warm, dry summers and autumns, and relatively cold winters with heavy snowfall in the interior; and in the north there is a continental climate with cold winters and warm, humid summers (48) (Figure 1).

### 2.2 *Culex pipiens* habitat suitability modeling and *Dirofilaria* spp. generations

*Culex pipiens* georeferenced points from Serbia were used from data previously obtained by Kurucz et al. (29), Kemenesi et al. (49) and Južnič-Zonta et al. (50). This mosquito species was selected for modeling as it is one of the most abundant species in Europe and has been reported as a vector of dirofilariosis in Serbia (7) and processed at a spatial resolution of 1 km^2^.

Environmental and bioclimatic variables were obtained in the same way as Rodríguez-Escolar et al. (42). In fact, 19 bioclimatic variables were downloaded from the World Clim website (51, 52) at a spatial resolution of 1 km^2^ for the years between 1970 and 2000 (current data), plus projected data for 2040, 2060, and 2080 (53). All variables were related to temperature and precipitation. Of the 19 bioclimatic variables, seven were selected taking into account a multicollinearity test performed in R based on Pearson’s correlation coefficient, in the same way as. In this study, variables with a cross-correlation coefficient *r* > ±0.75 were discarded and, according to vector biology, the following variables were selected: mean annual temperature (°C) (BIO_1_), isothermality (BIO_3_), seasonality of temperature (DE × 100) (BIO_4_), mean temperature of the wettest quarter (°C) (BIO_8_), mean temperature of the driest quarter (°C) (BIO_9_), annual precipitation (mm) (BIO_12_), *y* and seasonality of precipitation (coefficient of variation) (BIO_15_). In addition, five environmental variables (human footprint: built environment, population density, electric power infrastructure, cropland, grazing land, roads, railways and waterways (53), the presence of irrigated crop areas, the location of rivers and water bodies (54), and the density of shrubs and herbaceous plants (55) due to their effect on vector distribution) were selected.

To model the habitat suitability and geographic distribution of *Cx. pipiens* in the study area, the methodology of Morchón et al. (43) were used. In fact, we used the Maxent program (56) to calculate the habitat suitability of a species across environmental constraints (57). With the Kuenm package in R (58), the 119 best models generated in Maxent were chosen by combining a set of variables, 17 values of the regularization multiplier (0.1–1.0 at intervals of 0.1, 2–6 at intervals of 1, and 8 and 10), and the seven possible combinations of three feature classes (linear, quadratic, and product). The model performance was assessed in terms of statistical significance (Partial_ROC < 0.05), omission rates (OR = 5%), and model complexity using the Akaike information criterion corrected for small sample sizes (AICc). Significant models with an omission rate ≤ 5% were selected. Then, from this set of models, those with an AICc delta value of ≤2 were selected as the final candidate models. The candidate models were built using the “kuenm_cal” function, and the evaluation and selection of the best model were carried out using the “kuenm_ceval” function. Finally, the final ENM (best-fit model) was generated using the variables and the same parameters as previously selected. Ten bootstrap replications with logistic outputs were performed. The evaluation of these final models was based on the ROC_partial, OR, and AICc calculations using an independent dataset. The creation of the final models was carried out by using the “Kuenm_mod” function.

The number of annual *Dirofilaria* spp. generations was calculated using the model described by Genchi et al. (5, 39, 47), Rodríguez-Escolar et al. (42), and Morchón et al. (43) and in the R-software (v.4.3.0) with daily average temperature data between 1990 and 2016 in Serbia (59, 60). With this model, it is possible to quantify the complete development of microfilariae of *Dirofilaria* spp. up to larvae 3 within the culicid vectors (extrinsic incubation) where it is necessary to accumulate 130 growth degree days (GDD), in 30 days, at most, this number being the life expectancy of the culicid mosquito.

### 2.3 *Dirofilaria* spp. risk map and its validation

To obtain a risk map of *Dirofilaria* spp. in Serbia, we multiplied (weighting approach) the final ENM of *Cx. pipiens* and *Dirofilaria* spp. generations from the raster calculator in ArcMap 10.8. To validate the resulting *Dirofilaria* spp. risk map, points of presence of *D. immitis* and *D. repens* infected dogs were obtained from all over the country (17, 19–22, 24–26, 61–69) and overlaid on the risk map to see in which area they were living.

### 2.4 Forward projection and rank change analysis

To assess the potential effects of climate change on heartworm transmission risk dynamics, we employ the best performing *Cx. pipiens* model to extrapolate the bioclimatic variables analyzed for three different time periods: the 2040s (2021–2040), the 2060s (2041–2060), and the 2080s (2061–2080). Additionally, three different RCPs 8.5 scenarios were used with the HadGEM3-GC21-LL model (70). This model is one of the most widely used today to simulate the climate response to increasing greenhouse gas concentrations in Europe (71).

Once the estimates were made, it was necessary to determine the percentage of increase or decrease in suitable habitat for *Cx. pipiens* for Serbia. In fact, we convert the NEM and future projections into a binary map of presence and absence using the 10th percentile of the current model as a threshold. With the biomod2 script of the R program, a range shift analysis was performed to determine in which territories the greatest changes in *Cx. pipiens* distribution occur, as result of climate change, for the 2040, 2060, and 2080 scenarios compared to today (72).

## 3 Results

### 3.1 Habitat suitability model for *Culex pipiens*

**Figure 2:**
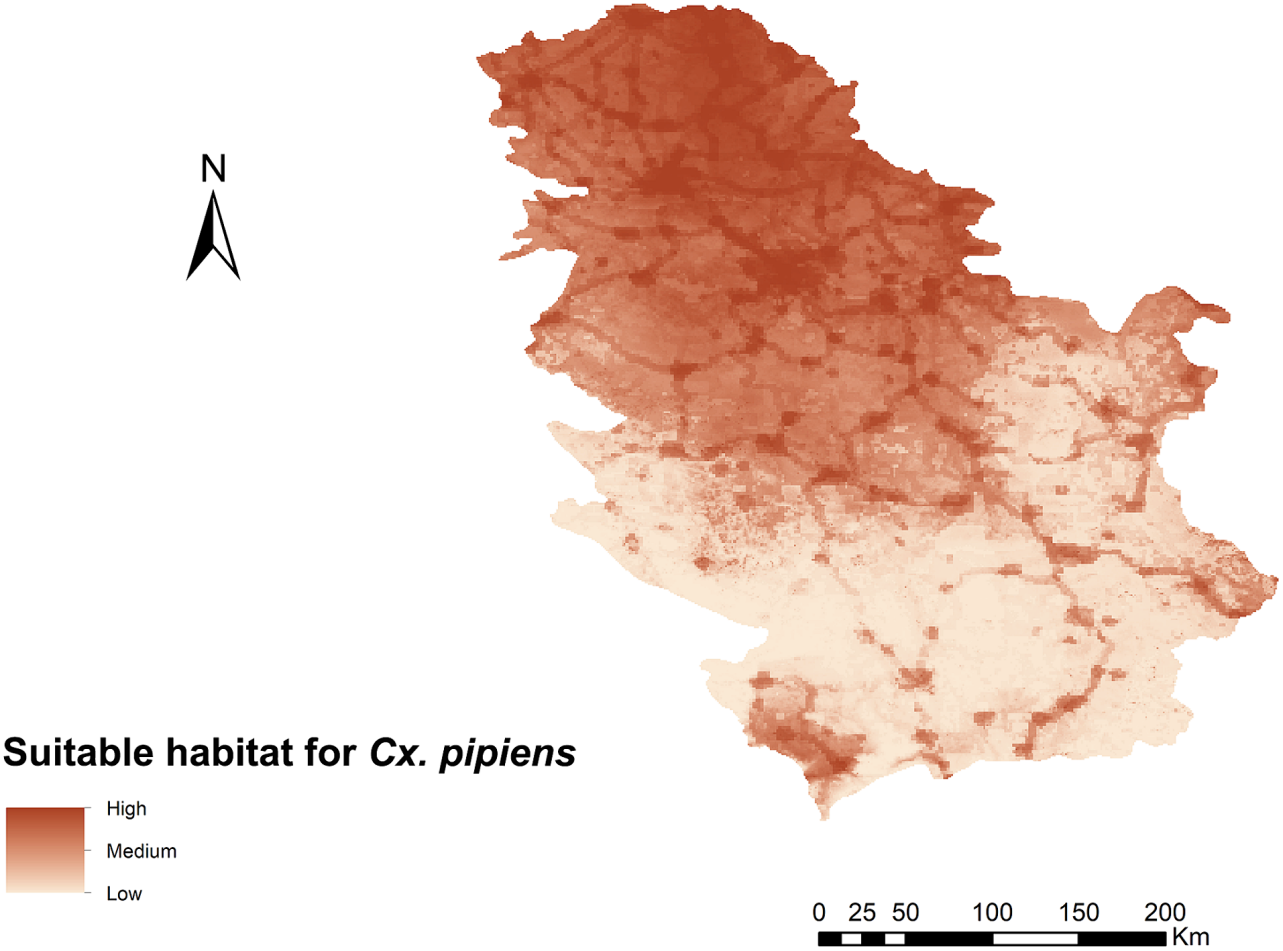
Ecological niche model for *Cx. pipens* in the geographical area of Serbia representing suitable habitat.

The curve value (AUC) of the *Cx. pipiens* ecological niche model for Serbia was 0.975, indicating very good predictive power. Habitat suitability for *Cx. pipiens* ranged from 0 to 0.93 (Figure 2), with the variables contributing most to the ENM Human footprint and BIO_15_ (Table 1). Of the 13 variables used, those with the highest contribution were the human footprint and BIO_15_ (Precipitation Seasonality) with a percentage contribution of 53 and 32.8%, respectively. The rest of the variables had lower values of 6.6%. Considering the map obtained, the area of highest habitat suitability for *Cx. pipiens* in Serbia is in the northern part of the country, an area that is part of the Pannonian plain with a larger human footprint and less mountainous than the south, where there is generally low suitability.

**Table 1:** Analysis of the contribution of the 13 environmental and bioclimatic variables to the ecological niche model for *Cx. pipiens*.

Variable	Percent contribution
Human footprint	53%
BIO_15_ (Precipitation seasonality)	32.8%
BIO_12_ (Annual precipitation)	6.6%
BIO_3_ (Isothermality)	4.7%
Rivers	1.4%
Herbaceous density	0.9%
Irrigated crops	0.3%
Water bodies	0.2%
BIO_1_ (Annual mean temperature)	0.1%
BIO_4_ (Temperature seasonality)	0%
Shrub density	0%
BIO_8_ (Mean temperature of wettest quarter)	0%
BIO_9_ (Mean temperature of driest quarter)	0%

### 3.2 Number *Dirofilaria* spp. generations

**Figure 3:**
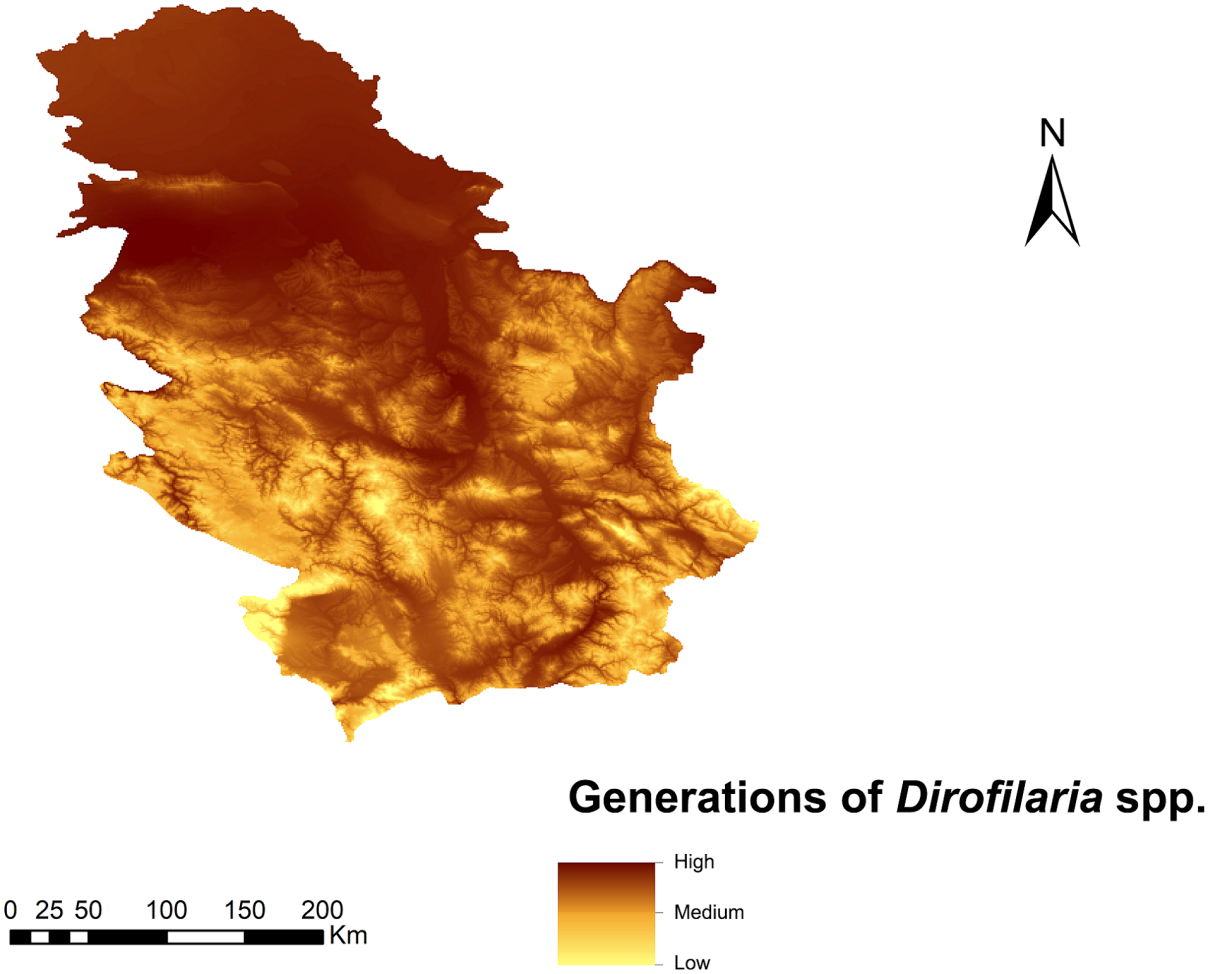
Prediction of the number of generations of *Dirofilaria* spp. in Serbia.

The highest value (>2.8) of the number of generations of *Dirofilaria* spp. was found in the Pannonian plain area (north of the country), where the number of generations is high due to the lower altitude (Figure 3). In the south, due to a more rugged orography, generations decrease with altitude (down to 0.09) except for the areas close to the main river basins.

### 3.3 Potential risk of transmission of *Dirofilaria* spp.

**Figure 4:**
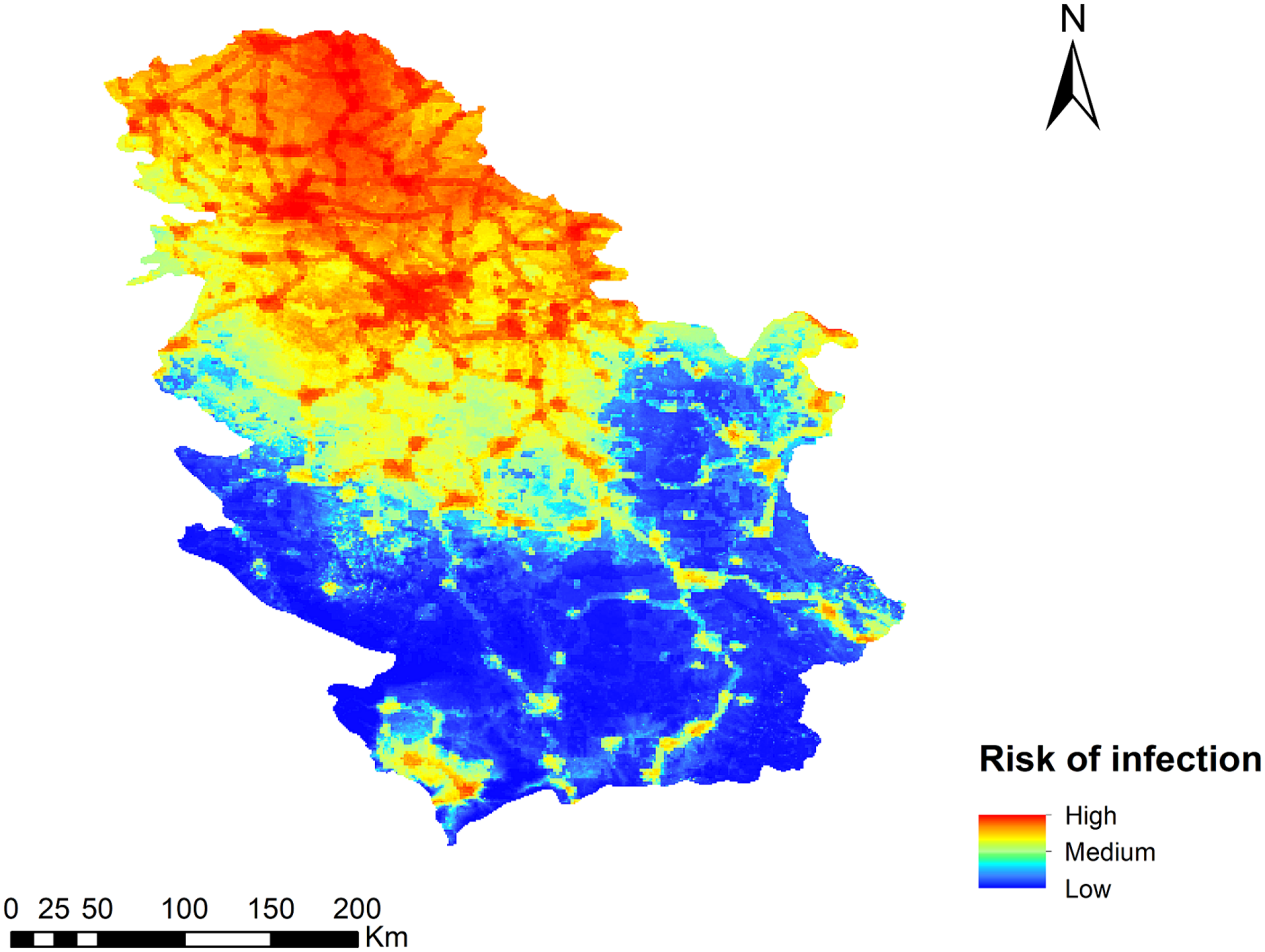
Ecological niche model of the risk of *Dirofilaria* spp. infection in Serbia.

The result of the *Dirofilaria* spp. transmission risk map in Serbia is shown in Figure 4. Generally speaking, the highest risk is found in the northern part of the country, decreasing as one moves toward the southern areas, with a more rugged relief and less human presence. In terms of territory, five ranges of values have been established (very high, high, medium, low, and very low), with 6.3 and 17.2% corresponding to very high and high risk areas respectively; 19.3% of the territory has a medium risk, 20.7% a low risk, and 36.5% a very low risk. The places where the risk of transmission is high or very high coincide with areas of low altitude, high human footprint and irrigated crops. In the south, the risk is generally low due to a more mountainous orography, with the exception of the basins of the main rivers as they are at a lower altitude.

**Figure 5:**
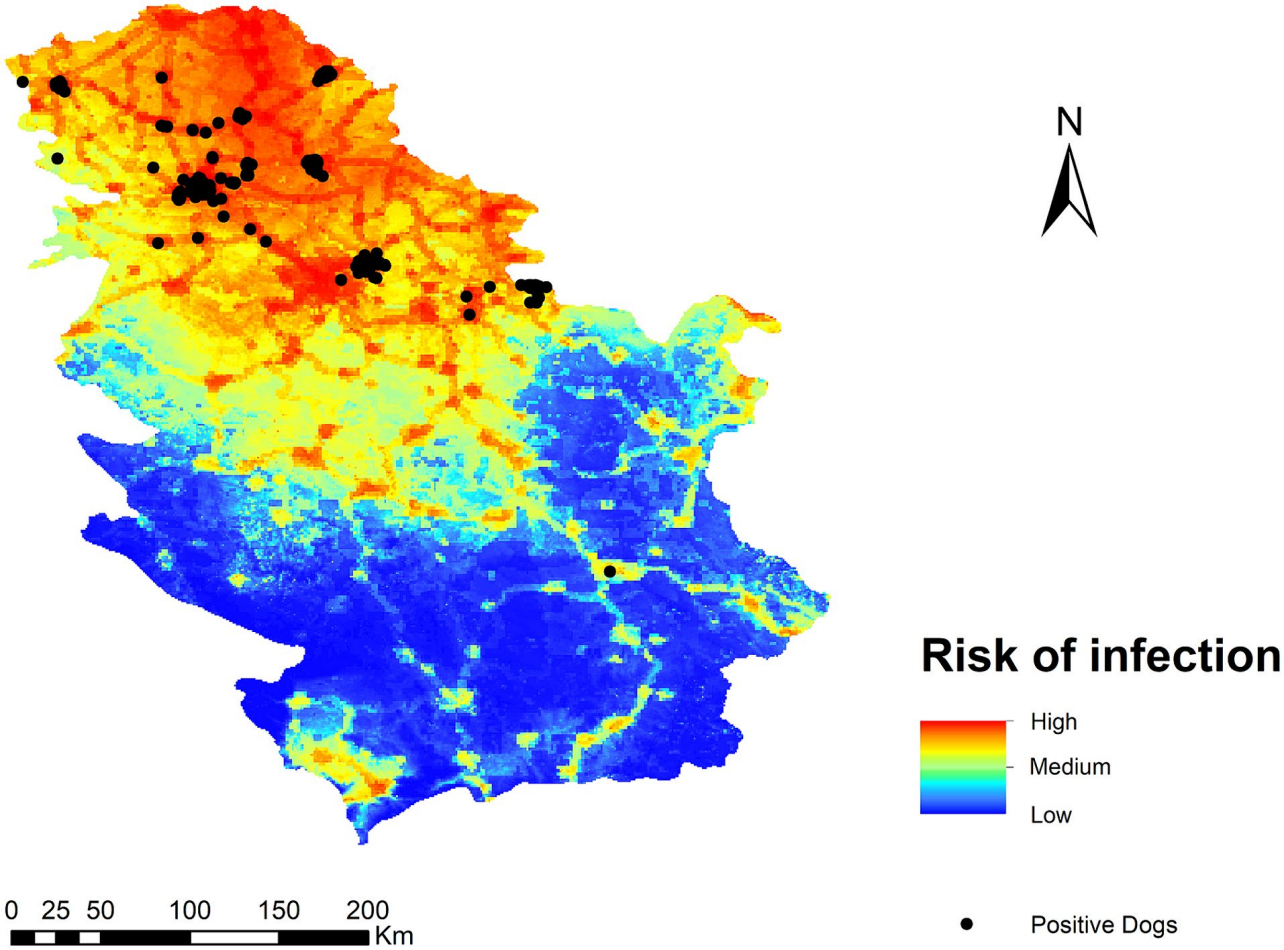
Ecological niche model of the risk of *Dirofilaria* spp. infection in Serbia with the locations of infected dogs according to Kurucz et al. (29), Kemenesi et al. (49), and Južnič-Zonta et al. (50).

To test our transmission risk map and validate it, geo-referenced points of *D. immitis* and *D. repens* infected dogs were superimposed. Of the *Dirofilaria* spp. positive dogs, 89.28% were found in very high-risk areas, 9.57% in high-risk areas, and 1.16% in moderate risk areas. In both low and very low risk areas, the percentage of positive dogs was 0% (Figure 5).

### 3.4 Future projection for the years 2040, 2060, and 2080 according to the climate change scenario RCP 8.5

**Figure 6:**
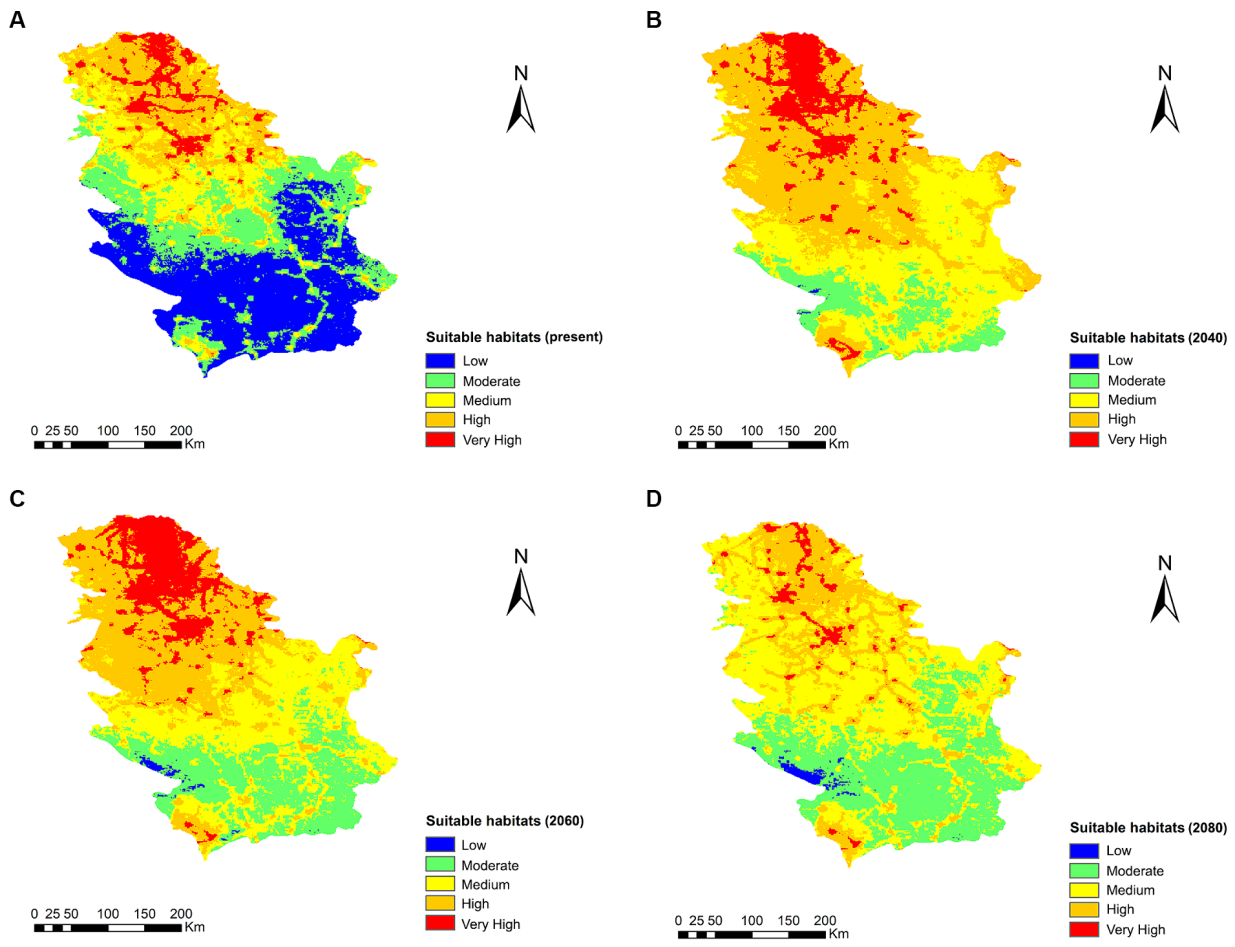
Suitable habitats for *Cx. pipens* at present **(A)** and their projections into the future, 2040 **(B)**, 2060 **(C)**, and 2080 **(D)**, in Serbia under the climate change scenario RCP 8.5.

The range change analysis shows a remarkable increase in the extent of suitable habitats for *Cx. pipiens* in 2040 and 2060, with the exception of 2080 where the change is very little appreciable (Figure 6). The percentage gain of territory for *Cx. pipiens* was 44.8% for 2040, 104.1% for 2060, and 2.9% for 2080. Notably, in 2080, there is a 65.7% percentage loss of suitable territory for the vector. Increases in areas suitable for the mosquito vector occur toward higher altitude areas in the south.

## 4 Discussion

Serbia in one of the countries in southeastern Europe where prevalences in infected dogs have continued to increase in recent years with ranges between 12.7 and 33.3%, mainly in the north of the country (20–25, 73) and where, for the first time, *Cq. richiardii*, *Cx. pipens*, and *Och. caspius* have been identified as vector species of the disease (29). This study is the first to map the risk of *Dirofilaria* spp. infection in Serbia using the distribution of the territory suitable for the survival of *Cx. pipiens*, one of the main and most abundant vectors of the disease in Europe (7), as well as including new predictor variables, and which has been validated using the presence of *Dirofilaria* spp. infected dogs as a reference. Within the biased spectrum of predictor variables that have been taken into account to date in most predictive models for Northeastern Europe (annual temperature records) (5, 39, 47, 74–77), in this study, we have incorporated several variables directly linked to the vector’s life cycle (humidity, rainfall, areas of naturally and/or artificially stagnant freshwater, rivers, density of herbaceous plants, irrigated agricultural areas, location of human populations, communications, agricultural activities, exchange of goods, and travel), as well as weighting with the number of generations of *Dirofilaria* spp. in the vector, with a robust and highly predictive result.

With the utilization of ecological niche modeling tools, it is possible to create risk models for zoonotic diseases that take into account a variety of abiotic variables regarding the development of a species, these tools predict the most likely habitats for the mosquitoes that carry the disease and have a high degree of resolution, even in areas where surveillance data are lacking (78). In South of Europe, a previous study has been utilized to validate the risk map associated with *Dirofilaria* spp. with the addition of the geolocation of infected animals, obtaining a higher resolution projection (1 km^2^) with a high significant and consistent (42, 43).

Genchi et al. (5) produced a map of the potential number of *Dirofilaria* spp. generations, where Serbia was located with average values, similar to those of the rest of central European countries, being higher in the north of the country. In our study, we have observed that the risk of infection by *Dirofilaria* spp. predominates in the north, which corroborates previous data, and centralizes the risk in places where human population, agricultural activity, and average rainfall are concentrated, these being the variables that contributed most to the model, suggesting the presence of *Cx. pipiens* is related to the presence of irrigated areas, a high density of human population and animals infected by *D. immitis* and/or *D. repens* and an increase in humidity. Moreover, if we take into account the wild carnivore population (7, 13, 24, 25, 27, 79–82) and others (82), our model increases in reliability as studies of *Dirofilaria* spp. infected animal populations show concentrated positivity, as well as infected domestic dogs, in the north of the country. There are also data from neighboring countries with high rates of *Dirofilaria* spp. infection such as Hungary, Romania, Bulgaria, Croatia, Bosnia, and Herzegoniva (7, 12, 15, 77, 83–90), which may increase the risk of infection.

The results of the 2040, 2060, and 2080 projections under climate change scenario RCP 8.5 revealed a displacement of the current distribution area of *Cx. pipiens* toward new territories, mainly in the south of the country, in where there is a significant potential increase in *Cx. pipiens* habitat, and therefore risk of infection, throughout the country and mainly in the south, with a 104.1% gain of ideal habitat for culicid vectors in 2060, although in 2080, there is a 65.7% percentage loss of suitable vector territory, decreasing in the north but remaining similar in the south. This is in line with other studies where there is an increase in temperatures, which is consolidated in areas with previously colder and in the future temperate climates, due to climate change and the transmission dynamics of certain vector-borne diseases (34, 42, 74, 90), therefore, from the point of view of One Health, measures should be taken by the Serbian government administration to take appropriate control measures and to interrupt the expansion and establishment of the vectors transmitting the disease.

In conclusion, this model will allow both health and veterinary scientists to diagnose the disease in previously unsuspected/clean areas, take more effective control measures, and further investigate the epidemiology of dirofilariosis in animals and humans. Consequently, disease alerts will be increased, considering each population’s specific situation. Further studies should be carried out to investigate the infection risk at a local level in order to take the necessary and optimal preventive measures to interrupt the spread of dirofilariosis in southern Europe in the coming years. Similar situations are already occurring in countries bordering Serbia, such as Croatia, Romania, Bulgaria, Hungary, and Greece. Thanks to this type of ecological niche model for *Cx. pipiens* and the prediction of the risk of infection for *Dirofilaria* spp., it will be possible to help health and veterinary personnel to carry out control measures both in areas where the disease is already diagnosed and in others where the health alert is lower. All of this will facilitate the action of veterinarians and doctors and the monitoring of the disease in specific locations in the country.

## Data availability statement

The raw data supporting the conclusions of this article will be made available by the authors, without undue reservation. All figures are originals created by the authors with Maxent and ArcMap 10.8 software.

## Author contributions

IR-E: Data curation, Formal analysis, Investigation, Methodology, Writing – original draft, Writing – review & editing. RH-L: Investigation, Software, Supervision, Validation, Writing – review & editing, Methodology. JS-A: Investigation, Software, Supervision, Writing – review & editing, Methodology, Validation. MC-C: Data curation, Formal analysis, Writing – review & editing. SS: Concep1tualization, Data curation, Investigation, Supervision, Validation, Writing – review & editing, Visualization. MŽ: Data curation, Investigation, Writing – review & editing. DM: Data curation, Investigation, Visualization, Writing – review & editing. RM: Conceptualization, Data curation, Funding acquisition, Investigation, Resources, Supervision, Writing – original draft, Writing – review & editing, Methodology, Validation, Visualization.
